# Cell-of-Origin Subtyping of Diffuse Large B-Cell Lymphoma by Using a qPCR-based Gene Expression Assay on Formalin-Fixed Paraffin-Embedded Tissues

**DOI:** 10.3389/fonc.2020.00803

**Published:** 2020-06-05

**Authors:** Wan-Hui Yan, Xiang-Nan Jiang, Wei-Ge Wang, Yi-Feng Sun, Yi-Xin Wo, Zheng-Zhi Luo, Qing-Hua Xu, Xiao-Yan Zhou, Jun-Ning Cao, Xiao-Nan Hong, Xiao-Qiu Li

**Affiliations:** ^1^Department of Pathology, Fudan University Shanghai Cancer Center, Shanghai, China; ^2^Department of Oncology, Shanghai Medical School, Fudan University, Shanghai, China; ^3^Canhelp Genomics Research Center, Hangzhou, Zhejiang Province, China; ^4^Department of Medical Oncology, Fudan University Shanghai Cancer Center, Shanghai, China

**Keywords:** diffuse large B-cell lymphoma, cell-of-origin, gene expression profiling, immunohistochemistry, quantitative polymerase reaction (PCR), formalin-fixed paraffin-embedded tissue

## Abstract

The well-established cell-of-origin (COO) algorithm categorizes diffuse large B-cell lymphoma (DLBCL) into activated B-cell-like (ABC) and germinal center B-cell-like (GCB) subgroups through gene expression profiling. We aimed to develop and validate a qPCR-based gene expression assay to determine the COO subgroups of DLBCL with formalin-fixed paraffin-embedded (FFPE) tissue. We first established a DLBCL transcriptome database of 1,016 samples retrieved from three published datasets (GSE10846, GSE22470, and GSE31312). With this database, we identified a qPCR-based 32-gene expression signature (DLBCL-COO assay) that is significantly associated with the COO subgroups. The DLBCL-COO assay was further validated in a cohort of 160 Chinese DLBCL patients. Biopsy samples from DLBCL patients with paired FFPE and fresh frozen tissue were collected to assign COO subtypes based on the immunohistochemistry (IHC) algorithm (Han's algorithm), DLBCL-COO assay, and global gene expression profiling with RNA-seq. For 111 paired FFPE and fresh DLBCL samples, the concordance between the IHC, qPCR, and RNA-seq methods was 77.5% and 91.9%, respectively. The DLBCL-COO assay demonstrated a significantly superior concordance of COO determination with the “gold standard” RNA-seq compared with the IHC assignment with Han's algorithm (*P* = 0.005). Furthermore, the overall survival of GCB patients defined by the DLBCL-COO assay was significantly superior to that of ABC patients (*P* = 0.023). This effect was not seen when the tumors were classified by the IHC algorithm. The DLBCL-COO assay provides flexibility and accuracy in DLBCL subtype characterization. These findings demonstrated that the DLBCL-COO assay might serve as a useful tool for guiding prognostic and therapeutic options for DLBCL patients.

## Introduction

Diffuse large B-cell lymphoma (DLBCL) is the most common subtype of malignant lymphomas, accounting for more than 40% of newly diagnosed cases. Although DLBCL is potentially curable with standard treatment, there is an urgent need for new therapies since most refractory or relapsed patients will eventually die from the disease. DLBCL has been recognized as a group of heterogeneous diseases with diverse genetic features and variable clinical outcomes. Almost two decades ago, Alizadeh et al. (1) performed gene expression profiling (GEP) with cDNA microarrays to explore unrecognized molecular heterogeneity in DLBCL. Using hierarchical clustering, there were at least two distinct groups within DLBCL: the germinal center B-cell-like (GCB) group and the activated B-cell-like (ABC) group. This method is widely recognized as the cell-of-origin (COO) classification algorithm. In a series of large randomized clinical studies following the establishment of COO classification, DLBCL patients with the ABC subtype showed significantly inferior characteristics compared with those with the GCB subtype, even in the clinical study evaluating the efficacy of immune chemotherapy (2).

In recent years, COO classification has been not only recognized as a prognostic factor but also used to tailor therapies for DLBCL patients (3). Additionally, COO classification or its surrogates are widely incorporated into the clinical development of state-of-the-art therapies for *de novo* and refractory/relapsed DLBCL patients (4). Thus, the World Health Organization (WHO) Classification for Lymphoid Malignancies required the determination of COO for every newly diagnosed DLBCL case. However, COO classification using cDNA microarrays or RNA-seq is not economical or flexible for surgical pathology laboratories and is not compatible with formalin-fixed, paraffin-embedded (FFPE) samples. Immunohistochemistry (IHC) panels, such as the Hans algorithm, may be applied as surrogates and are widely used, but there is low concordance with cDNA microarray or RNA-seq classification, and intraobserver and interobserver variation may undermine their accuracy (5). Although medium-throughput assays, such as NanoString, may be applied to FFPE samples and may be accurate compared with the “gold standard” assay (6), the integrated and enclosed platform, high price, and sophisticated workflow may limit their routine application.

In the current study, we developed a novel gene expression assay (DLBCL-COO assay) that allows differentiation between the GCB and ABC DLBCL subtypes in FFPE specimens using a quantitative reverse transcription polymerase chain reaction (qPCR) platform and evaluated the DLBCL-COO assay against RNA-seq and IHC assays. We further discussed its potential application in routine clinical practice as well as the clinical development of novel therapies for DLBCL patients.

## Materials and Methods

### Gene Expression Database Curation

The DLBCL gene expression datasets with confirmed COO subtypes were collected from a public data repository, the National Center for Biotechnology Information (NCBI) Gene Expression Omnibus (GEO) database, and curated to form a comprehensive DLBCL transcriptome database. The gene expression datasets retrieved from three GEO series (GSE10846, GSE22470, and GSE31312) were mainly conducted on two different Affymetrix oligonucleotide microarray platforms, including the GeneChip Human Genome U133A Array and the U133 Plus 2.0 Array. Detailed descriptions of the specimen characteristics and clinical features are provided in the original studies.

### Gene Expression Data Analysis

Normalization and analysis of gene expression data were performed using R software and packages available from the Bioconductor project (www.bioconductor.org). The single-channel array normalization (SCAN) approach from the SCAN-UPC package was used to process the Affymetrix microarray data (7, 8). Upon normalizing each raw CEL file, SCAN outputs probe-level expression values. We further used custom mapping files from the BrainArray resource to summarize the probe-level intensities directly to gene-level expression values (9). Thus, probe mapping to multiple genes and other problems associated with older generations of Affymetrix probe designs were avoided. After normalization, we applied the ComBat approach to adjust for batch effects (10). To identify a gene expression signature, we used the recursive feature elimination–support vector machine (RFE-SVM) algorithm for feature selection and classification modeling (11). A linear SVM classifier was derived using the training samples with known ABC or GCB labels and applied to the test samples. When the probability predicted by the DLBCL-COO assay that a sample belongs to the ABC or GCB subgroup is >75%, the specimen is classified as the ABC or GCB subtype accordingly. Otherwise, specimens with a probability lower than 75% were considered unclassified. The Database for Annotation, Visualization and Integrated Discovery (DAVID) bioinformatics resource was used to integrate functional genomic annotations (12). A biological network was constructed by NetworkAnalyst software (www.networkanalyst.cn, version 3.0) (13, 14). Protein–protein interactions were retrieved from the IMEx Interactome Database (15).

### Development of the DLBCL-COO Assay

The DLBCL-COO assay was developed on the Applied Biosystems 7500 Real-Time PCR system (Applied Biosystems, Foster City, CA, USA), targeting 32 candidate markers and three housekeeping genes identified with microarray analysis. To support clinical applications using FFPE samples with poor RNA quality, primers were designed to amplify short template mRNA regions of exon-spanning junctions. In addition, the TaqMan MGB probes incorporate a 5′ fluorescent reporter dye and a 3′ nonfluorescent quencher, which offers the advantage of lower background signal, resulting in better precision in quantitation.

### Case Selection

The study was approved by the ethical committee of Fudan University Shanghai Cancer Center (Approval case number: 1904199-18). Specimens between June 1, 2012, and December 19, 2018, that were maintained at the Department of Pathology, Fudan University Shanghai Cancer Center, were retrospectively archived in the current study. All cases were reviewed by two independent pathologists (X-NJ and X-QL) in compliance with WHO classification and were histologically confirmed as *de novo* DLBCL, not otherwise specified (DLBCL, NOS).

### Morphology and Immunohistochemistry

The following antibodies (Ventana Medical Systems, Tucson, Arizona, USA) were applied on a BenchMark XT automated immunostainer (Ventana Medical Systems, Tucson, Arizona, USA) with Cell Conditioning 1 heat retrieval solution (Ventana Medical Systems, Tucson, Arizona, USA): anti-CD10, anti-BCL6, and anti-MUM1. For all staining procedures, tonsils with reactive hyperplasia served as external controls and reactive lymphocytes as internal controls. The cut-off value for tumor positivity was set at 30% of tumor cell staining for CD10, BCL6, and MUM1. Cases were designated as GCB or non-GCB using the algorithm specified by Hans et al. (16). The morphological and IHC results were independently evaluated by two pathologists (W-HY and X-QL).

### Sample Processing and qPCR Analysis

Total RNA was isolated from FFPE tissue and fresh tissue using the RecoverAll Total Nucleic Acid Isolation Kit (Thermo Fisher Scientific, Waltham, MA, USA) per the manufacturer's guidelines. The concentration of total RNA was quantified by a Qubit 3.0 Fluorometer (Thermo Fisher Scientific, Waltham, MA, USA), while RNA integrity and quality were further appraised using agarose gel electrophoresis. For each sample, reverse transcription was performed on isolated total RNA using the High-Capacity cDNA Reverse Transcription Kit with RNase Inhibitor (Applied Biosystems, Foster City, CA, USA). The PCR program consisted of an initiation step at 95°C for 10 min, followed by 40 cycles at 95°C for 15 s and 60°C for 1 min. All measurements were taken in triplicate. The melting curves of each measurement were checked; only the coordinate results were included in the subsequent analysis. Three genes (IPO8, PGK1, and TFRC) that have been reported to be consistently expressed in DLBCL cells were selected as housekeeping genes. First, qPCR results of housekeeping genes with various sample storage duration and RNA quality were investigated, and then, the average Ct value of each target gene minus the mean of three housekeeping genes was calculated as ΔCt. The –ΔCt value of each gene was applied for downstream analysis.

### RNA Sequencing and Data Analyses

RNA-seq was performed on the NovaSeq 6000 system (Illumina, San Diego, CA, USA) using 1 μg of RNA extracted from fresh tumor tissue according to the manufacturer's instructions. The raw sequencing data were preprocessed using the BRB-SeqTools suite (https://github.com/DeplanckeLab/BRB-seqTools). The GEP-based classification method was performed to determine the COO molecular subtype of each specimen as described in Wright et al. (17) and Reddy et al. (18).

### Statistical Analysis

For comparison with the Hans-based IHC method, all COO subtypes of samples from the GEP methods were categorized as either “GCB” or “non-GCB.” All GCB predictions remained GCB, and any “ABC” or “UNC” subtype predictions from the RNA sequencing and qPCR assays were converted to the “non-GCB” subtype. The concordance between any pair of assays was calculated using only the total number of samples that could be called by both of those assays. The overall percent agreement and asymptotic 95% confidence intervals (CIs) are presented. To determine the positive percent agreement (PPA) and negative percent agreement (NPA), the global GEP-based subtyping method served as a standard reference in each comparison. Overall survival (OS) was defined as the time from diagnosis until death or the last follow-up date. Kaplan–Meier survival curves were constructed for OS analysis. A value of *p* = 0.05 was determined as the level of statistical significance. All statistical analyses were performed using SPSS Statistics version 23 software (SPSS Inc., Chicago, USA).

## Results

### Establishment of the DLBCL Transcriptome Database

**Figure 1:**
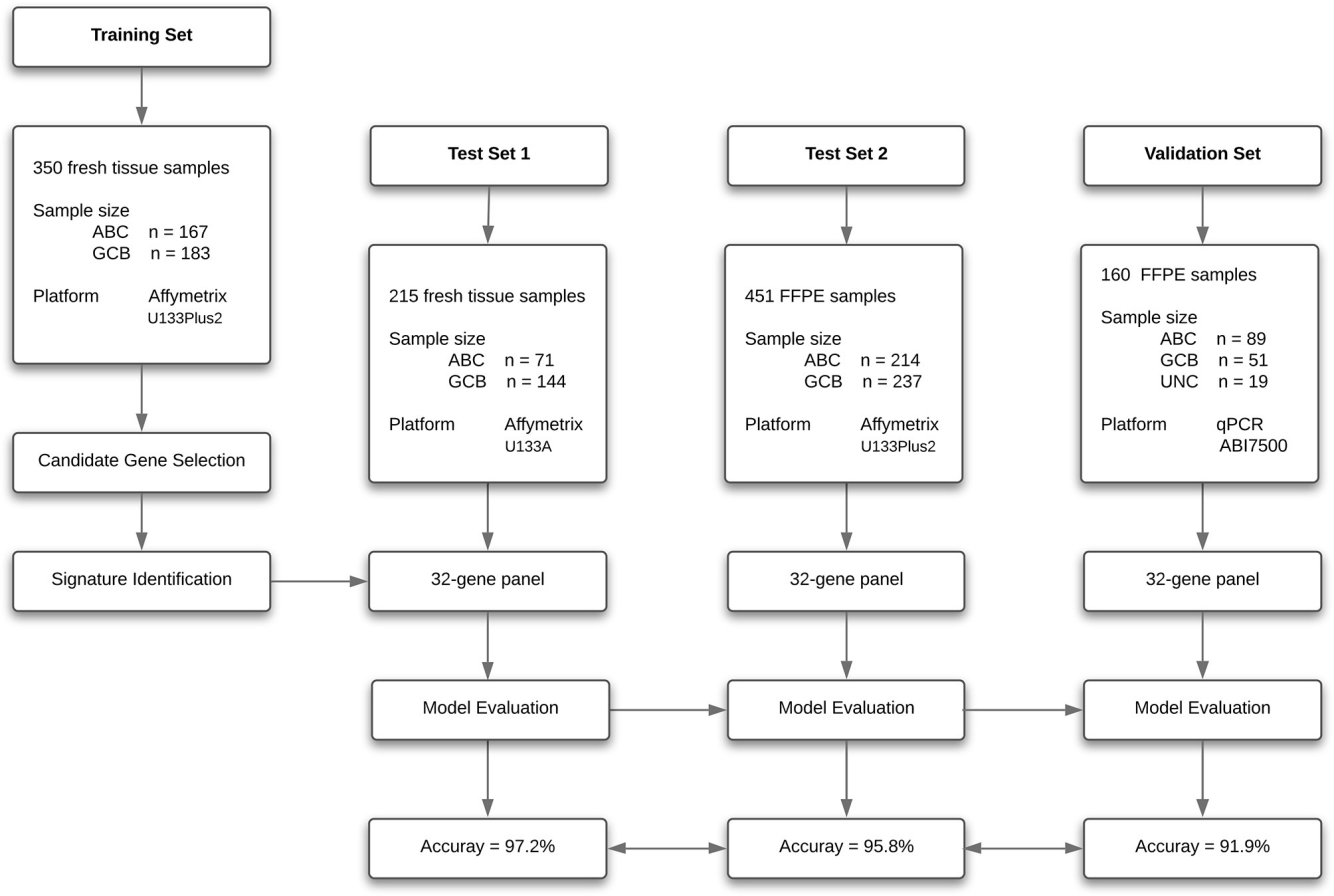
Flow diagram of gene expression signature identification and performance assessment.

To create a DLBCL transcriptome database for COO subtype classification, we performed a systematic search of major biological data repositories [e.g., ArrayExpress, GEO, and The Cancer Genome Atlas (TCGA)] to collect gene expression data sets of DLBCL samples with a confirmed COO subtype status. Overall, we accumulated the gene expression profiles of 1,016 samples to form a comprehensive DLBCL transcriptome database. We further adopted a training–testing–validation approach to identify and validate a reliable gene expression signature in this study. First, the gene expression profiles of 167 ABC samples and 183 GCB samples were retrieved from the database and curated to form a training set. Second, two independent cohorts were used as *in silico* test sets to evaluate the classification performance: one was composed of the gene expression profiles of 215 fresh tumor samples (Test Set 1, 71 ABC samples and 144 GCB samples), and the other was composed of the gene expression profiles of 451 FFPE samples (Test Set 2, 214 ABC samples and 237 GCB samples). Third, the developed qPCR assay was clinically validated against the RNA-seq and IHC assays on paired fresh and FFPE samples of *de novo* DLBCL patients treated at Fudan University Shanghai Cancer Center (Validation Set). Figure 1 depicts the three distinct phases of our study design, and Tables 1, 2 summarize the clinical characteristics of the samples in the study.

**Table 1:** Summary of sample information.

**Samples**	**Training Set**	**Test Set 1**	**Test Set 2**
Tissue type	Fresh frozen tissue	Fresh frozen tissue	Formalin-fixed paraffin-embedded (FFPE)
No. of patients	350	215	451
Cell-of-origin (COO) subtypes (%)			
Activated B-cell-like (ABC)	167 (47)	71 (33)	214 (47)
Germinal center B-cell-like (GCB)	183 (53)	144 (67)	237 (53)
Data source	GSE10846	GSE22470	GSE31312
Platform	Affymetrix Human Genome U133 Plus 2.0 Array	Affymetrix Human Genome U133A Array	Affymetrix Human Genome U133 Plus 2.0 Array

**Table 2:** Clinical characteristics of diffuse large B-cell lymphoma (DLBCL) patients stratify by immunohistochemistry (IHC) and DLBCL-COO.

**Variable and level**	**IHC (*****n*** **=** **160)**			**DLBCL COO (*****n*** **=** **159)**			
	**GCB**	**non-GCB**	***P*-value**	**GCB**	**ABC**	**Unclassified**	***P*-value**
	**(*n* = 60)**	**(*n* = 100)**		**(*n* = 51)**	**(*n* = 89)**	**(*n* = 19)**	
**Gender**			0.838				0.809
Male	32 (53)	55 (55)		27 (53)	49 (55)	10 (53)	
Female	28 (47)	45 (45)		24 (47)	40 (45)	9 (47)	
**Age**			**0.045**				0.137
Median	58 (23–81)	62 (26–81)		58 (23–79)	62 (34–81)	61 (26–78)	
≤ 60	35 (58)	42 (42)		29 (57)	39 (44)	9 (47)	
>60	25 (42)	58 (58)		22 (43)	50 (56)	10 (53)	
**Clinical stage**			**0.002**				**0.001**
I–II	43 (72)	46 (46)		37 (73)	39 (44)	13 (68)	
III–IV	17 (28)	54 (54)		14 (27)	50 (56)	6 (32)	
**ECOG**			0.092				0.327
0–1	59 (98)	91 (91)		49 (96)	81 (91)	19 (100)	
2–4	1 (2)	9 (9)		2 (4)	8 (9)	0 (0)	
**Serum LDH**			**0.035**				0.141
Normal	42 (70)	52 (53)		34 (67)	48 (54)	12 (66)	
Elevated	18 (30)	46 (47)		17 (33)	41 (46)	6 (33)	
Missing	0	2		0	0	1	
**Extranodal site**			**0.014**				**0.003**
≤ 1	53 (88)	68 (72)		45 (90)	57 (67)	18 (95)	
>1	7 (12)	27 (28)		5 (10)	28 (33)	1 (5)	
Missing	0	5		1	4	0	
**IPI**			**0.004**				**0.003**
0–1	39 (65)	41 (44)		33 (66)	35 (41)	12 (66)	
2	16 (26)	21 (23)		13 (26)	20 (24)	4 (22)	
3	4 (7)	18 (19)		2 (4)	19 (22)	1 (6)	
4–5	1 (2)	13 (14)		2 (4)	11 (13)	1 (6)	
Not calculable	0	7		1	4	1	

ECOG, Eastern Cooperative Oncology Group; LDH, lactate dehydrogenase; IPI, International Prognostic Index. *P-values in bold font indicate the differences were statistically significant.

### Identification of a 32-Gene Expression Signature in the Training Set

The training set consisted of 167 ABC and 183 GCB samples. After the data normalization and annotation steps, a matrix of 20,342 unique genes in 350 samples (≈7.12 million data points) was prepared for downstream bioinformatics analyses. Extracting a subset of informative genes from high-dimension genomic data is a critical step for gene expression signature identification. Here, we deployed the RFE-SVM algorithm with the linear SVM classification model and the parameter C equal to 1. The algorithm identified a compact panel of 32 genes that are significantly associated with the two molecular subtypes. As listed in Table 3, 16 genes were overexpressed in the ABC subtype, and 16 genes were overexpressed in the GCB subtype. We further investigated whether the 32 candidate genes exhibited biological features relevant to the DLBCL molecular subtypes. As shown in Table 4, the most significantly enriched gene categories are involved in B-cell differentiation, B-cell activation, humoral immune response, and hemopoiesis. We also explored the underlying biological networks of the selected candidate genes. We used the 32 genes as seeds to generate a minimum protein–protein interaction network. The network comprised 21 genes of the 32-gene set and was centered on essential nodes such as *BCL6, UBC, AICDA, LMO2, UCHL1*, and *MME* (Figure S1).

**Table 3:** Description of 32 gene annotation.

**Gene Symbol**	**Gene Description**	**Cytoband**	**Subtype**
GPR183	G protein-coupled receptor 183	13q32.3	ABC
IGHM	Immunoglobulin heavy constant mu	14q32.33	ABC
P2RX5	Purinergic receptor P2X 5	17p13.2	ABC
CCL22	C–C motif chemokine ligand 22	16q21	ABC
FCMR	Fc fragment of IgM receptor	1q32.1	ABC
SH3BP5	SH3 domain-binding protein 5	3p25.1	ABC
JADE3	Jade family PHD finger 3	Xp11.3	ABC
BATF	Basic leucine zipper ATF-like transcription factor	14q24.3	ABC
BLNK	B-cell linker	10q24.1	ABC
DOCK10	Dedicator of cytokinesis 10	2q36.2	ABC
AICDA	Activation-induced cytidine deaminase	12p13.31	ABC
TOX2	TOX high mobility group box family member 2	20q13.12	ABC
MIR155HG	MIR155 host gene	–	ABC
NLRP7	NLR family pyrin domain containing 7	19q13.42	ABC
FAM129C	Family with sequence similarity 129 member C	19p13.11	ABC
MPEG1	Macrophage expressed 1	11q12.1	ABC
BCL6	B-cell CLL/lymphoma 6	3q27.3	GCB
CR2	Complement C3d receptor 2	1q32.2	GCB
LMO2	LIM domain only 2	11p13	GCB
LRMP	Lymphoid restricted membrane protein	12p12.1	GCB
MME	Membrane metalloendopeptidase	3q25.2	GCB
MYBL1	MYB proto-oncogene like 1	8q13.1	GCB
RGS13	Regulator of G protein signaling 13	1q31.2	GCB
TUBB2A	Tubulin beta 2A class IIa	6p25.2	GCB
UCHL1	Ubiquitin C-terminal hydrolase L1	4p13	GCB
XIST	X inactive specific transcript (non-protein coding)	Xq13.2	GCB
CILP	Cartilage intermediate layer protein	15q22.31	GCB
CD83	CD83 molecule	6p23	GCB
STAG3	Stromal antigen 3	7q22.1	GCB
RGCC	Regulator of cell cycle	13q14.11	GCB
VPREB3	V-set pre-B cell surrogate light chain 3	22q11.23|22q11	GCB
HOPX	HOP homeobox	4q12	GCB

**Table 4:** Enrichment analysis of 32 genes (*P* < 0.05).

**Term**	***P*-value**	**Count**	**Genes**
B-cell differentiation	6.16E−05	4	GPR183, BCL6, AICDA, BLNK
B-cell activation	2.43E−04	4	GPR183, BCL6, AICDA, BLNK
Humoral immune response	2.72E−04	4	CD83, GPR183, CR2, BLNK
Hemopoiesis	5.16E−04	5	GPR183, LMO2, BCL6, AICDA, BLNK
Lymphocyte differentiation	5.94E−04	4	GPR183, BCL6, AICDA, BLNK
Immune response	6.36E−04	7	CD83, GPR183, CCL22, CR2, AICDA, IGHM, BLNK
Hemopoietic or lymphoid organ development	7.42E−04	5	GPR183, LMO2, BCL6, AICDA, BLNK
Immune system development	9.28E−04	5	GPR183, LMO2, BCL6, AICDA, BLNK
Leukocyte differentiation	1.19E−03	4	GPR183, BCL6, AICDA, BLNK
Lymphocyte activation	3.93E−03	4	GPR183, BCL6, AICDA, BLNK
Leukocyte activation	6.77E−03	4	GPR183, BCL6, AICDA, BLNK
Cell activation	1.08E−02	4	GPR183, BCL6, AICDA, BLNK
Defense response	1.61E−02	5	NLRP7, CD83, CCL22, CR2, BLNK
Regulation of CD4-positive, alpha beta T-cell differentiation	2.41E−02	2	CD83, BCL6
Regulation of alpha-beta T-cell differentiation	3.99E−02	2	CD83, BCL6
Negative regulation of cell differentiation	4.75E−02	3	LMO2, HOPX, BCL6
Negative regulation of signal transduction	4.95E−02	3	CILP, BCL6, RGS13

### Independent Validation in Fresh and FFPE DLBCL Samples

The classification model comprising 32 subtype-specific genes was established using the entire training set and then applied to Test Set 1, which was composed of 71 ABC and 144 GCB fresh frozen samples. With the 32-gene expression signature, 69 samples were classified as ABC and 146 as GCB. The overall agreement between the 32-gene expression signature classification and the reference diagnosis reached 97.2% (209 of 215; 95% CI: 0.94–0.99). The PPA was 98.6% (142/144, 95% CI: 0.95–1.00), and the NPA was 94.4% (67/71, 95% CI: 0.85–0.98), considering GCB as positive (Table 5). It was of interest to evaluate the classification performance of the gene expression signature in FFPE samples. We further applied the 32-gene expression signature to Test Set 2, which was composed of 214 ABC and 237 GCB FFPE samples. Of the 451 samples, the 32-gene expression signature classified 239 as ABC and 212 as GCB. The agreement between the gene expression-based assignments and the reference diagnoses reached 93.6% (422 of 451; 95% CI: 0.909–0.957). The PPA was 88.6% (210/237, 95% CI: 0.84–0.92), and the NPA was 99.1% (212/214, 95% CI: 0.96–1.00), considering GCB as positive (Table 5).

**Table 5:** Overall concordance between DLBCL-COO assay and reference diagnosis in two test sets.

**DLBCL-COO Assay**	**Reference Diagnosis**		
	**GCB**	**ABC**	**Subtotal**
**Test Set 1**			
GCB	142	4	146
ABC	2	67	69
Subtotal	144	71	215
**Test Set 2**			
GCB	210	2	212
ABC	27	212	239
Subtotal	237	214	451

### Clinical Validation of the 32-Gene Expression Signature by qPCR Analysis

**Figure 2:**
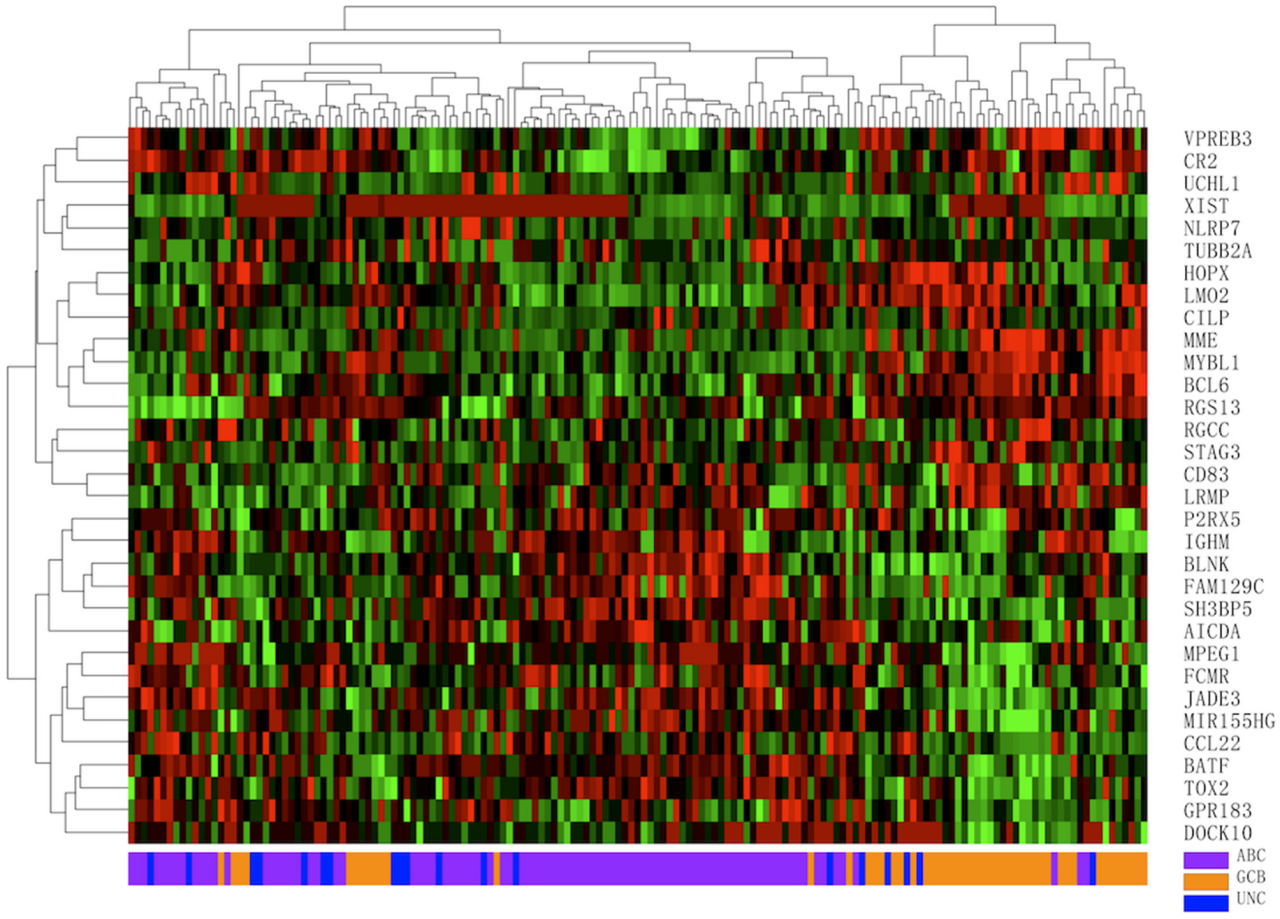
Hierarchical clustering analysis of 32 gene expression profiles in 159 diffuse large B-cell lymphoma (DLBCL) specimens. Colored pixels capture the magnitude of the expression for each gene, where shades of red and green represent over-expression and under-expression, respectively. Right: the official symbol of 32 genes; left: a dendrogram of hierarchical clustering of these genes. Bottom: COO subgroups including germinal center B-cell-like (GCB, orange), activated B-cell-like (ABC, purple), unclassified (UNC, blue); top: a dendrogram of hierarchical clustering of all samples.

A total of 160 DLBCL patients with confirmed COO subtypes based on IHC assignment were enrolled in the current study. Han's algorithm assigned 60 cases (37.5%) as GCB and 100 cases (62.5%) as non-GCB. One hundred fifty-nine of 160 FFPE specimens met all criteria and were successfully assayed by the DLBCL-COO assay. We first evaluated the hierarchical clustering of the 32 genes and 159 samples based on the qPCR data. Complete linkage hierarchical clustering analysis was performed where the metric of similarity was Pearson's correlation between the 32-gene expression profiles of the samples. As shown in Figure 2, the samples were clustered into distinct groups that followed the COO subtypes. Among the three subtypes, most GCB samples clustered together, whereas the unclassified samples were more similar to ABC samples. According to the predictions by the 32-gene signature, 89 cases (56.0%) were classified as ABC, 51 cases (32.1%) as GCB, and 19 cases (11.9%) as unclassified. In addition, 113 DLBCL patients had paired fresh frozen tissue, and 111 cases passed stringent quality control for RNA-seq analysis. The gold standard RNA-seq method defined 34 cases (30.6%) as GCB, 50 cases (45.1%) as ABC, and 27 cases (24.3%) as unclassified. The concordance between DLBCL-COO and RNA-seq and the concordance between IHC Han's algorithm and RNA-seq are summarized in Table 6. The DLBCL-COO assay demonstrated a significantly superior concordance of COO determination with the gold standard RNA-seq compared with the IHC assignment with Han's algorithm (91.9 vs. 77.5%; *P* = 0.005). Additionally, the PPA and NPA of the DLBCL-COO assay assigning GCB/non-GCB were 88.2% (30 of 34, 95% CI: 0.72–0.96) and 93.5% (72 of 77, 95% CI: 0.85–0.98), respectively.

**Table 6:** Overall concordance between methods of identifying subtypes of DLBCL in validation set.

**Validation Set**	**RNA-seq**		
	**GCB**	**non-GCB**	**Subtotal**
**DLBCL-COO assay**			
GCB	30	5	35
Non-GCB	4	72	76
Subtotal	34	77	111
**IHC (Han's algorithm)**			
GCB	25	16	41
Non-GCB	9	61	70
Subtotal	34	77	111

**Figure 3:**
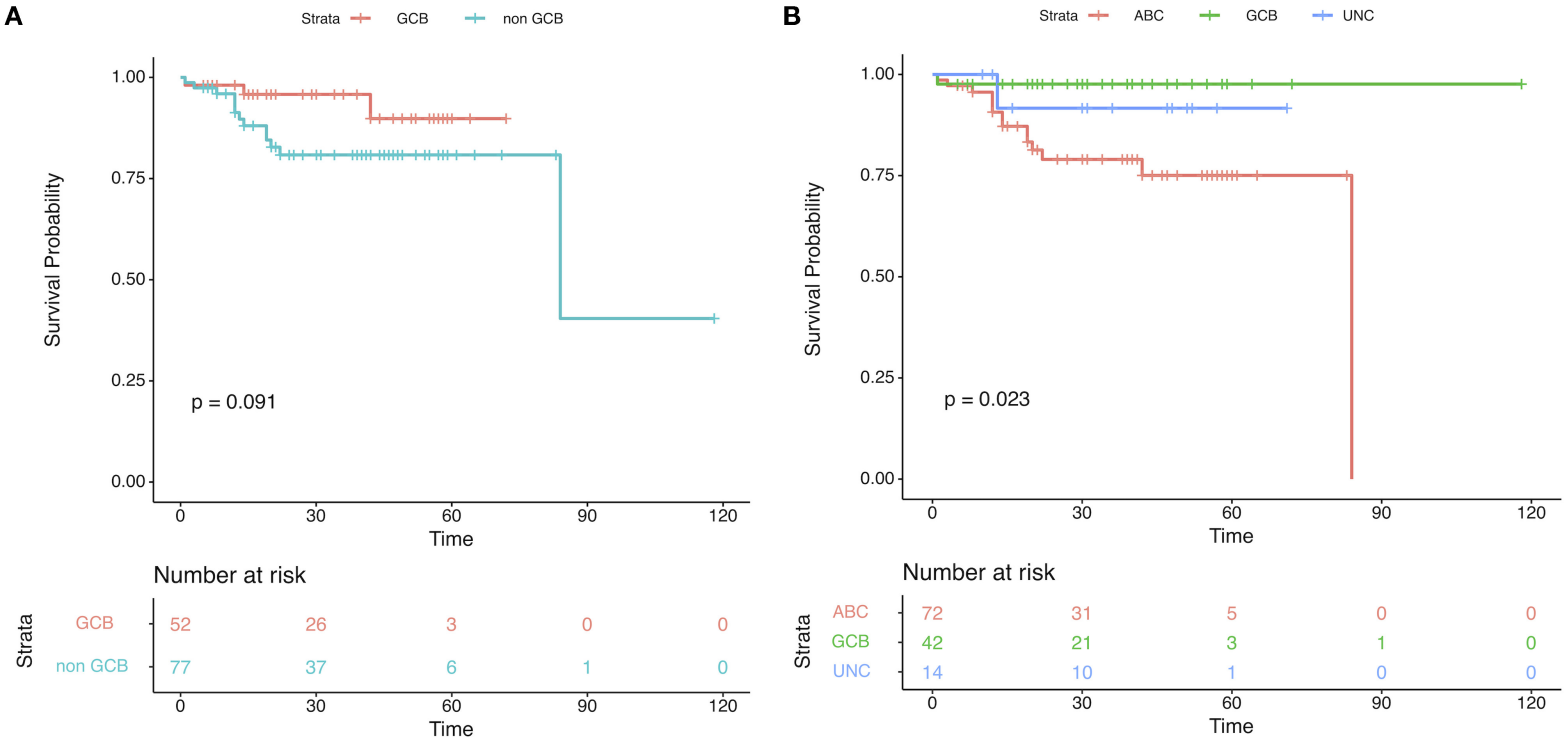
Patient outcomes according to cell-of-origin (COO) in the validation set. Kaplan–Meier plots of overall survival based on molecular subgroups stratified by **(A)** immunohistochemistry (IHC) and **(B)** DLBCL-COO assay. *P*-values were obtained using the log-rank test. UNC, Unclassified.

One hundred twenty-nine DLBCL cases with survival information, IHC assignment results, and DLBCL-COO assay results were identified. The clinical information related to the IHC and DLBCL-COO assignment results is summarized in Table 2. Han's algorithm failed to stratify DLBCL patients, mostly treated with the R-CHOP (rituximab, cyclophosphamide, doxorubicin, vincristine, and prednisone) regimen, into different prognostic groups (Figure 3A) (*P* = 0.091). However, the OS of GCB patients defined by the DLBCL-COO assay was significantly superior to that of ABC patients (Figure 3B) (*P* = 0.023). The patients assigned as unclassified DLBCL had an intermediate OS (Figure 3B).

## Discussion

As DLBCL is a heterogeneous disease in genetic, biological, and clinical behavior, precise classification is critical for predicting prognosis or the efficacy of therapies. Characterizing DLBCL into GCB and ABC based on COO represents a milestone in the heterogeneity delineation of DLBCL. These COO classification results successfully correlated with the patient outcome, even in the era of immunochemotherapy with rituximab (2). The COO classification system demonstrated the different cancer biology and etiologies in DLBCL, making it possible to tailor therapies to different subgroups of patients. The most exciting application of COO classification may be the efficacy prediction of BTK inhibitors and lenalidomide in treating refractory or relapsed DLBCL patients (19, 20). Although randomized phase three clinical studies evaluating the efficacy of BTK inhibitors and lenalidomide in treating treatment-naive DLBCL based on the COO classification failed (21, 22), COO determination for newly diagnosed DLBCL patients is still mandatory. Several novel classification systems based on the DLBCL genetic landscape have been proposed recently, like four genetic subtypes based on the status of MYD88 L265P, CD79b mutations, NOTCH1 mutations, BCL6 fusion, NOTCH2 mutations, BCL2 translocations, and EZH2 mutations (23). However, these systems highly interacted with the COO classification (23, 24), indicating that COO classification may be the backbone of other state-of-the-art classification algorithms.

As the gold standard of COO determination is global GEP based on cDNA microarray or RNA-seq, which is inaccessible for routine testing, the most widely used and flexible COO surrogate is IHC. However, the interobserver and intraobserver reproducibility of IHC COO assignment are not satisfactory, and the concordance across different IHC COO algorithms is quite low (25). The IHC COO assignment failed to predict the outcome of DLBCL patients treated with immunochemotherapy (26) and failed to predict the efficacy of the BTK inhibitor ibrutinib in treating DLBCL (27). Several medium through-put assays compatible with FFPE samples have been developed in recent years, demonstrating high concordance with global GEP based on cDNA microarray or RNA-seq (6). Nonetheless, the complexity of the assay based on a specific platform (NanoString) or the Illumina sequencer and the high cost may potentially limit its wide application in routine practice, especially in poor resource areas.

Therefore, it is necessary to establish a COO determination assay with appropriate cost, comparable accuracy with the gold standard assay, and compatibility with FFPE samples. The qPCR technology is generally considered the “gold standard” procedure for measuring individual gene expression and is often used to confirm the findings of DNA microarray and RNA-seq analyses. Furthermore, the qPCR technology can be easily applied to FFPE specimens, and thus, it is widely applicable in clinical practice. Recently, Tekin et al. reported a successful validation of a qPCR-based six-gene predictor for DLBCL prognosis in an international clinical study (28). Herein, the DLBCL-COO assay is a qPCR assay that detects a 32-gene expression profile for DLBCL molecular classification. The DLBCL-COO assay was trained against the so-called gold standard of COO assignment using GEP on fresh frozen tissue, tested, and then validated in multiple independent cohorts. Although a slight loss in signal intensities was observed when FFPE sample storage duration increased (Figure S2), the qPCR-based TaqMan assays remained accurate and robust for gene expression profiling. The overall successful rate of the DLBCL-COO assay is satisfactory (159/160, 99%), even for the FFPE samples archived 5 years ago, indicating satisfactory compatibility with FFPE samples. This may be critical for relapsed or refractory DLBCL, as biopsied samples may be archived for several years. Regarding accuracy, the concordance of the DLBCL-COO assay with the gold standard RNA-seq assay was 91.9%, which was comparable to the NanoString and HTG assays, even though there is a lack of head-to-head studies, suggesting that the COO assignment by DLBCL-COO is precise.

In addition to routine clinical practice, the clinical development of novel therapies for DLBCL also requires a COO assignment assay with high accuracy and consistency, and short turnover duration. In the PHOENIX study, which is a randomized, double-blind, placebo-controlled, multicenter, phase 3 study comparing the efficacy and safety of ibrutinib in combination with R-CHOP vs. placebo in combination with R-CHOP in patients with the newly diagnosed non-GCB subtype of DLBCL, GEP showed that 75.9% of patients with non-GCB DLBCL assigned by IHC had ABC DLBCL (23). As central pathology COO assignment and review were applied in this well-controlled study, the concordance between the IHC COO assignment and GEP assignment may be much lower. In another phase 3 study evaluating the efficacy of R-CHOP plus lenalidomide in previously untreated ABC DLBCL (ROBUST study), the NanoString Lymph2Cx GEP assay was applied to assign COO, demonstrating 15% failure in the test (29). As the samples from previously untreated DLBCL patients were recently biopsied in the ROBUST study, the failure rate may be higher for the long-archived samples of relapsed and refractory DLBCL patients. In these settings, a more compatible assay beyond GEP as well as a more accurate assay beyond IHC will be more effectively incorporated into clinical development.

## Conclusion

In conclusion, the DLBCL-COO assay provides flexibility and accuracy in DLBCL subtype characterization into GCB and ABC. These subtype distinctions should help guide disease prognosis and treatment options in DLBCL clinical practice. Further prospective studies including incorporation into prospective interventional studies will be needed to evaluate the performance in detail.

## Data Availability Statement

Publicly available datasets were analyzed in this study, these can be found in the NCBI Gene Expression Omnibus (GSE10846, GSE22470, GSE147986, and GSE31312).

## Ethics Statement

The studies involving human participants were reviewed and approved by the Clinical Research Ethics Committee of Fudan University Shanghai Cancer Center. The patients/participants provided their written informed consent to participate in this study.

## Author Contributions

W-HY, X-NJ, W-GW, and X-QL designed the study. W-HY, X-NJ, W-GW, Y-FS, Y-XW, and Z-ZL performed the experiments. W-HY, Y-FS, and Q-HX analyzed all data. W-HY, X-NJ, W-GW, and Y-FS wrote the initial manuscript draft. Q-HX, X-YZ, J-NC, X-NH, and X-QL critically revised the manuscript and gave valuable insight to the study concept. All authors revised the manuscript and read and approved the final manuscript.

## Conflict of Interest

Y-FS, Y-XW, Z-ZL and Q-HX are employed by Canhelp Genomics Co., Ltd. The authors declare that this study received funding from Canhelp Genomics Co., Ltd. The funder had the following involvement with the study: data collection and analysis, and preparation of the manuscript. The remaining authors declare that the research was conducted in the absence of any commercial or financial relationships that could be construed as a potential conflict of interest.
