# Supplementary figures and images for: Cell-of-Origin Subtyping of Diffuse Large B-Cell Lymphoma by Using a qPCR-based Gene Expression Assay on Formalin-Fixed Paraffin-Embedded Tissues
Source: Front Oncol. 2020 Jun 5;10:803. doi: 10.3389/fonc.2020.00803 (PMC7292205; doi:10.3389/fonc.2020.00803)

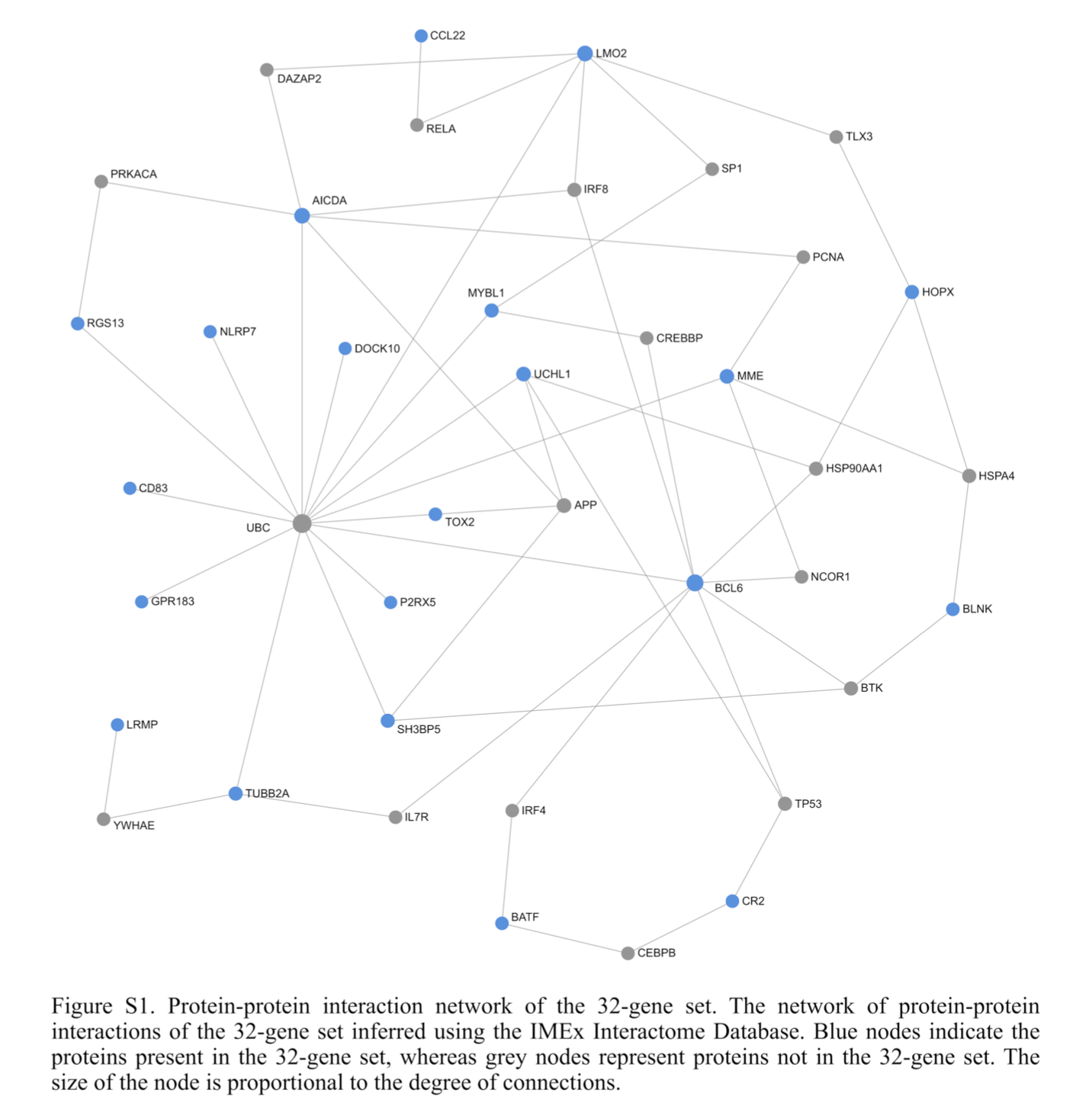

Supplement: Supplementary file 1 [file Image_1.PNG]

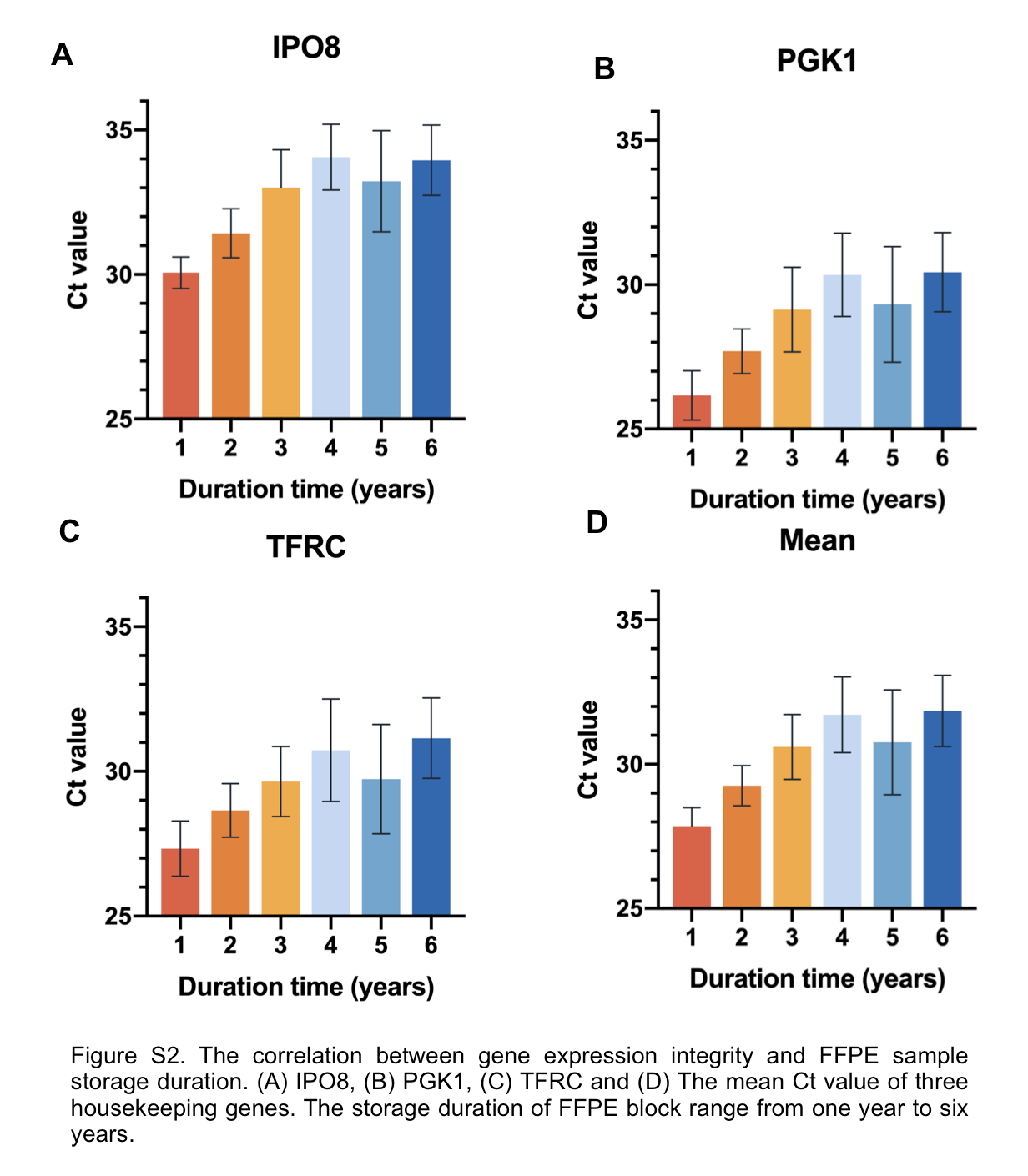

Supplement: Supplementary file 2 [file Image_2.PNG]
